# Patient perceptions of nurse mentors facilitating the Aussie Heart Guide: A home‐based cardiac rehabilitation programme for rural patients.

**DOI:** 10.1002/nop2.34

**Published:** 2015-10-04

**Authors:** Terence John Frohmader, Frances Lin, Wendy Chaboyer

**Affiliations:** ^1^ Department of Intensive Care MedicineLaunceston General HospitalLauncestonTasmaniaAustralia; ^2^ School of Nursing and Midwifery – Centre for Health Practice Innovation (HPI)Griffith UniversityGold Coast CampusQueenslandAustralia; ^3^ NHMRC Centre of Research Excellence in NursingCentre for Health Practice InnovationMenzies Health Institute QueenslandGriffith UniversityGold Coast CampusQueenslandAustralia

**Keywords:** Aussie Heart Guide, cardiac rehabilitation, coronary heart disease, home‐based cardiac rehabilitation, mentoring, myocardial infarction, nurses, nursing

## Abstract

**Aim:**

To explore and describe long‐term thoughts and perceptions of the Aussie Heart Guide Programme including the role of the mentor, held by patients recovering from myocardial infarction.

**Design:**

A qualitative design.

**Methods:**

Thirteen patients recovering from myocardial infarction who were unable to attend a hospital‐based or affiliated outpatient cardiac rehabilitation programme were interviewed by telephone at the completion of the programme and asked to describe the relationship with their assigned nurse mentor and their perception of the audiovisual used in the programme.

**Results:**

Three themes emerged; assisting me to cope, supporting me and my family and tailoring the programme to my needs. Patients were satisfied with the programme and appreciative of the supportive and caring relationships provided by mentors during their hospitalization through to their discharge from the programme.

## Introduction

Cardiac rehabilitation (CR) is a validated, evidenced‐based service (Leon *et al*. 2005) offered to people who have experienced a cardiac event such as myocardial infarction (MI) or at risk of developing coronary heart disease (CHD). CR describes: ‘all measures used to help people with heart disease return to an active and satisfying life and to prevent reoccurrence of cardiac events’ (National Heart Foundation of Australia & Australian Cardiac Rehabilitation Association 2004, p1). Principally, CR services are provided in hospital and community settings, run by health professionals and depending on the setting, usually in a multidisciplinary framework and guided by best practice guidelines that feature core components (Thomas *et al*. 2007). Frequently these services are not provided in rural and remote settings (De Angelis *et al*. 2008) due to a lack of resources such as appropriate clinicians living in the areas (Mason 2013) and distances between small communities making the services less economically viable. With CR being integral to the comprehensive long‐term cardiac care of patients diagnosed with CHD (World Health Organization Committee 1993), people living in these rural and regional communities potentially miss out on this vital component of their care (Dollard *et al*. 2004) after being diagnosed with CHD.

Several innovative CR programs have been proposed to meet the needs of patients who cannot access traditional hospital‐based CR programs (Clark *et al*. 2013) and examples include home‐based self‐help manuals (Lothian Primary Care Trust 2002) and various examples of telehealth‐based models of CR delivery (Neubeck *et al*. 2009). Home‐based CR programs are usually delivered to patients in their home (Blair *et al*. 2011) and when compared with hospital‐based CR, patient outcomes including mortality, cardiac events, exercise capacity, modifiable risk factors and health‐related quality of life have not been (Taylor *et al*. 2010) found to be statistically different (Dalal *et al*. 2007). Apart from complimenting hospital‐based CR programs, they have been found to be safe (Blair *et al*. 2011) and cost effective (Clark *et al*. 2013). Importantly, home‐based CR programs appear to offer patients choice in terms of participating in CR and therefore widen its accessibility for patients unable to undertake hospital‐based programs due to transport issues and financial constraints associated with living remotely. A recent meta‐analysis comparing home‐based CR to hospital‐based CR (Clark *et al*. 2010) found that while results were promising, the effectiveness of home‐based CR was unclear due to the small number of trials that were short in duration, methodologically of poor to moderate quality and the wide variety of complex interventions used to deliver CR interventions. However, several studies have found home‐based CR programs delivered by telephone to be as effective as hospital‐based CR programs in terms of patient outcomes and cost (Blair *et al*. 2011). Participants of these studies were generally satisfied with the psychological support, lifestyle advice and education provided by health professionals using this mode of service delivery (Vale *et al*. 2003, Hanssen *et al*. 2007, Hawkes *et al*. 2013, Wakefield *et al*. 2014).

A recent systematic review suggests further research is needed to determine the effectiveness of home‐based CR on cardiac events leading to readmission to hospital and its cost, uptake, use and completion by rural cardiac populations (Clark *et al*. 2013). Currently there is insufficient evidence to support the use of specific models of home‐based CR due to the lack of research undertaken in remote populations (Clark *et al*. 2013). In recognition of this, the Aussie Heart Guide Programme (AHGP) has been proposed to meet the CR needs of people diagnosed with heart disease in remote areas of Australia. This self‐help styled programme is introduced to patients recovering from MI prior to leaving hospital. The programme uses written and DVD based education materials and is facilitated by CR health practitioners who had some basic training in cognitive behavioural change techniques which included motivational interviewing, discussion of patient health beliefs, goal setting designed to promote self efficacy, reduce their risk of further problems, improve physical functionality and quality of life. Referred to as nurse mentors in this study, these professionals worked closely with patients to ascertain their health needs then set health goals which were recorded in a patient diary. Mentors contacted patients by telephone regularly to check progress and to monitor their rehabilitative path. Goals commonly included a graduated walking programme, dietary modification, smoke cessation, stress management and relaxation. To date, there has been limited evaluation of this new programme, thus the aim of this study was to explore and describe long‐term thoughts and perceptions of the AHGP including the role of the mentor, held by patients recovering from MI.

## Methods

### Design

This paper is part of an unpublished larger case study where the structures, processes and outcomes of the AHGP were examined in a single, explanatory case study (Yin 2009). As a methodology, case studies are useful because they involve robust, in‐depth investigations of single or multiple phenomena that may include an individual, group or organization (Schneider & Whitehead 2013) and can be used to evaluate programs (Baxter & Jack 2008); for this reason it was an appropriate methodology to use in this research. As part of the case study, interviews with participants who completed the AHGP were undertaken.

### Setting and participants

Study participants were adults (18 years or older), recovering in hospital from MI and live remotely in rural communities. Patients who had previously consented to undertake AHGP were invited to participate in the study. Participants were telephoned by the researcher on completion of their AHGP and asked if they would like to volunteer to undertake a telephone interview. All patients chose to complete the interview at the first point of contact. Purposive sampling was undertaken to enrich data and reflect patient interest in wishing to share their experiences and variation in terms of gender, marital and work status and time since completion of the AHGP. The inclusion and exclusion criteria relative to the study participants are reproduced in Table 1. Patients who were unwilling for whatever reason (distance to existing CR services, cost associated with travel, no available transport) to attend a hospital‐based CR programme were invited to participate in the evaluation of the AHGP and signed a consent form.

**Table 1:** Inclusion and exclusion criteria for study participants

Inclusion criteria	Exclusion criteria
Adults Confirmed MI Reside in non‐metropolitan areas of Tasmania Able to read and write in English Not cognitively impaired	Clinically unstable (due to uncontrolled arrhythmia, unstable angina or heart failure [class 3 or 4]) A history of major psychiatric illness (including dementia). Other significant co‐morbidities that may preclude the ability to exercise Severe eyesight or hearing impairment which would prevent the reading of or listening to the AHG resources.

### The intervention

The AHGP was adapted from the *Heart Manual* a home‐based CR programme developed in the United Kingdom (Lewin *et al*. 1992) and modified for the Australian setting by a committee of CR practitioners from the Australian Cardiovascular Health and Rehabilitation Association in 2008. The AHGP is an audiovisual resource facilitated by nurse ‘mentors’ providing CR to people who are unable to attend hospital‐based CR programme via telephone calls. After meeting with participants in hospital, mentors routinely contacted their patients within 1–2 days of leaving hospital and then weekly for approximately 6 weeks to provide individualized guidance, support and clarification of any issues arising from discharge.

### Data collection

Despite being used widely in quantitative research, telephone interviews are not commonly used in qualitative research (Novick 2008) with preference often given to face‐to‐face interviewing. Some believe that telephone interviewing produces inferior data compared with other data collection methods. However, this assertion is not supported by research evidence (Novick 2008) and importantly, telephone interviewing has several advantages compared with other interviewing techniques. They are considered less threatening to participants especially when sensitive information is being sought, do not rely on reading or writing skills compared with surveys and are more feasible when participants are dispersed geographically (Bourque & Fielder 2003, Opdenakker 2006). Telephone interviews are also cost effective, less time consuming to conduct (Opdenakker 2006), discourage feelings of social pressure and may improve researcher/participant rapport (Novick 2008). Disadvantages of telephone interviewing include the absence of visual cues (Opdenakker 2006), timing of the interview, cost of long distance phone charges and the need for participants to have a telephone (Bourque & Fielder 2003).

Participants enrolled in the study were interviewed by the researcher via telephone over a two week time period. AHGP completion for interviewed participants ranged from 6 months–2 years. The interviews were semi‐structured, ranged between 10–30 minutes in duration and notes were written during patient responses and then typed on to a MS Word document. A core aspect of the AHGP was mentoring, which became one focus of the interview. The interview guide consisted of three broad questions: ‘What was your impression of the AHGP?’ ‘What did having a nurse mentor mean to you during your recovery?’ and ‘What was good or bad about the mentor?’ Prompts were used to elicit more information from participants as required.

### Ethical considerations

Prior to enrolling in the study, potential participants were given oral and written information about the study. They were advised that their names would remain confidential, with no identifying data used in any reports or publications. They were told if they choose to withdraw at any time, their care would not be affected and that their data would be stored securely, according to national standards. If they agreed to participate, they signed a consent form at the start of the study. Verbal consent was reaffirmed from each participant prior to conducting the telephone interview component of the study. The study conforms to the principles outlined in the Declaration of Helsinki (1964). Written ethical approval to conduct this study was provided by the Human Research Ethics Committee of Griffith University (NRS/02/10/HREC) and the Human Research Ethics Committee (Tasmania) Network (H0009996). Written consent was obtained from all participants.

### Data analysis

**Figure 1:**
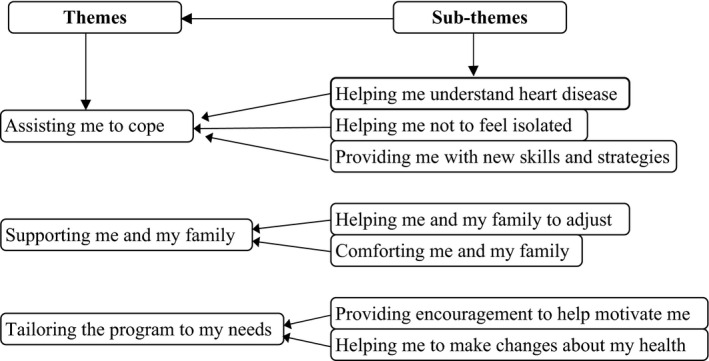
Themes and sub‐themes emerging from the patient interviews.

Thematic analysis (Braun & Clarke 2006) was used to analyse the data. All narrative transcripts were read repeatedly to achieve immersion and a sense of the participant's unique and complex perspective of two broad domains (mentoring and the AHGP). Codes were assigned to participants’ verbatim statements. After coding, key thoughts, patterns and conceptualizations emerged from the data inductively (Boyatzis 1998), leading to the grouping and labelling of sub‐themes which were repeatedly rechecked and questioned and classified into a hierarchal set of overarching themes relative to each domain. Frequent recursive and iterative discussion among the research team ensured that the codes, sub‐themes and emergent themes accurately reflected and encompassed the data (Figure 1).

An issue of trustworthiness of findings in qualitative data analysis is often considered in relation to credibility, dependability, transferability and confirmability (Lincoln & Guba 1985). In the current study, purposive sampling (Roberts & Burke 1989) ensured a broad representation of patients and analyst triangulation (Patton, 2001) occurred from regular meetings with the research team who ensured codes, sub‐themes and final themes accurately reflected the data for transferability, credibility and confirmability. A code book and memos were written to document the analytic process including decisions about emerging sub‐themes and final themes. An audit trail (Lincoln & Guba 1985) of the analysis also enhanced confirmability.

## Findings

A total of 13 phone interviews were undertaken, comprising of seven female and six male participants. Ages of the participants ranged from 46–69 years with the majority living between 50–100 kilometres from the nearest hospital‐based outpatient CR programme. Characteristics of the patients who participated in the telephone interviews are detailed in Table 2. Findings from the patient telephone interviews revealed several subthemes which were coded, organized and presented in three themes (Figure 1): assisting me to cope, helping me to understand heart disease and tailoring the programme to my individual needs. The first and second themes reflected participants’ focus on mentors with fewer comments about the AHGP itself.

**Table 2:** Sample characteristics of the participants

Characteristics	Patients (*n *=* *13)	
	Male	Female
	$\overset{¯}{X}$/sd	$\overset{¯}{X}$/sd
Age (in years)	60·8/11·61	58·5/10·8
Gender	7	6
Marital status		
Married/partner	5	3
Lives alone	2	3
Employment		
Yes	4	4
No	1	–
Disability	1	1
Retired	1	1
Distance to CR		
<50 km	2	2
50–100 km	4	3
>100 km	1	1

CR, cardiac rehabilitation; km, kilometres; $\overset{¯}{}$, Mean; SD, standard deviation.

### Assisting me to cope

Participants described how mentors assisted them to cope with the initial shock of having a heart attack and the period of adjustment following hospital discharge and returning home. Mentors provided assistance by listening to their concerns, explaining hospital procedures, providing information about cardiac disease, treatment and practical support in terms of organizing an individualized plan to follow after discharge from hospital. Participants were appreciative when mentor support was empathic, reassuring, recognized individuality, were respectful and afforded them the opportunity to regain a sense of control in their care. The sub‐themes detailed in this theme include; ‘helping me understand heart disease’; ‘helping me not to feel isolated’; and ‘providing me with new skills and strategies’.

In the sub‐theme ‘helping me understand heart disease’, it was common for participants to have little understanding of CHD in general and specifically, its cause, treatment and impact on their future. Mentors were instrumental in providing the information participants needed to understand the serious implications of heart disease and what aspects of their lifestyle they may need to change to reduce the risk of future cardiac events. They achieved this by providing practical explanations, using integrative teaching aids and highlighting aspects of the written programme resources applicable to the practical needs of each individual patient:

She told me what I needed to know and then it was up to me to do it. I do not think she could have done more. She explained everything in an easy way. (Participant 9)

The sub‐theme ‘helping me not to feel isolated’, accentuated a common emotion expressed by participants who lived alone or were isolated from medical services. Feelings of isolation, according to some patients, were exemplified by the uncertainty that a similar episode may occur again or a further cardiac event may prove fatal. Participants found regular mentor contact helped alleviate some of the emotional discomfort associated with social isolation:

I live a long way from the city. I do not go to the local doctor much. I am very isolated. The mentor helped me cope after being discharged from hospital. (Participant 13)

The final sub‐theme, ‘providing me with new skills and strategies’, involved the importance participants attributed to learning about CHD in collaboration with their mentors and acquire practical skills to enhance their ability to develop self‐care structures. Participants commonly expressed the importance of establishing a good relationship with their mentor to make decisions relevant to achieving their health goals. One participant stated; ‘Her phone contacts were greatly appreciated and I think it kept me on track in terms of eating better and exercising each day’ (Participant 5).

In terms of strategies to assist in their recovery and future personal health plans, participants believed that mentor efforts to provide them with an individualized plan of care was integral in terms of actively participating in their own care post hospitalization. Strategies such as goal setting and weekly diary planning provided participants with a flexible but structured approach in terms of their recovery. One participant stated:

She helped me get ready to leave the hospital and told me about what I had to do to get better and what to do each day. We put stuff in my diary so I wouldn't forget what I had to do. (Participant 11)

### Supporting me and my family

This theme highlighted the importance participants placed in their mentors providing ongoing support to them while in hospital and after discharge. Importantly, this also included mentors providing support for family members and involving them in discussions about their enrolment in the AHGP and planning of care following hospital discharge. In situations where patients and their families were emotionally overcome by the seriousness of their hospitalization, participants stated mentors were able to provide information and support in an encouraging way so as to promote acceptance of their heart condition and recovery. The sub‐themes described within the theme providing support to me and my family include; ‘helping me and my family to adjust’; and **‘**comforting me and my family’.

The sub‐theme ‘helping me and my family to adjust’ identifies that the mentors helped them when they were in deep personal stress and attempting to come to terms with their illness and its effect on partners and family. Some participants had an expectation that mentors would help them through a period of self adjustment by providing information and support to them and loved ones which would assist them in their homecoming. Participants claimed that mentors were empathetic to family concerns and issues and were proficient at clarifying issues relating to care in the weeks following discharge:

My partner was really upset and beside himself. He thought I could die at any moment. I had to send him home from the hospital because he was going to pieces. She [the nurse mentor] took him away and spent a good while with him discussing everything and going through the programme and recovery. This action really turned things around for us. (Participant 4)

In the sub‐theme ‘comforting me and my family’, participants commonly felt mentors provided them with emotional support during their recovery. Support was forthcoming in several ways. Mentors were thought to embrace and understand many of the difficulties faced by many patients experiencing a life changing event. Mentors who were empathetic, listened intently and considered their concerns, tailored a plan of care with them and who gave positive encouragement were identified as nurses keenly interested in them as a person. Mentors who were cheerful, friendly, hopeful and positive in terms of patient outlook were appreciated:

I was really down in the dumps for about 3 months after my heart attack. I'm not sure if it was because I went through a lot in hospital or because of my age but I found the mentor phone calls to help somewhat. (Participant 13)

### Tailoring the programme to my needs

The theme tailoring the programme to my needs identified the importance participant's placed on being treated holistically as individuals. Feelings of individualism appeared to be influenced by the participant's level of knowledge regarding CHD disease and their emotional reaction to it often appeared linked to their heterogeneity, age, gender, culture and social backgrounds. Participants responded positively to mentors who tailored their recovery in consideration of their individual needs. Sub‐themes for this theme include; ‘providing encouragement to help motivate me’ and ‘helping me to make changes about my health’.

The sub‐theme ‘providing encouragement to help motivate me’ highlighted the often convoluted nature of participant recovery from MI and the importance of mentors providing timely and ongoing encouragement during the programme. Participants sometimes found it difficult to meet their health goals due to physical setbacks, fatigue and negative emotions. Some suggested they were more likely to be motivated to meet their health goals if they perceived their mentor to be interested in them as a ‘person:

She encouraged us [participant and partner] to do the home rehab programme together, to motivate us I think. She was big on having the desire to get up and get going again and that is what we have done. (Participant 2)

In the sub‐theme ‘helping me to make changes about my health’ participants believed mentor advice and support positively influenced their recovery path by helping them understand the need to change unhealthy lifestyle behaviours. In addition, the written resources generally assisted them to understand the need to adopt a healthy lifestyle to lessen the risk of complications or future cardiac events. One participant explained; ‘She said if I worked hard at improving some things like regular walking, giving up cigarettes and looking after myself better, that I should have a full recovery’(Participant 1). While comments about mentors were generally positive, one participant did not think the mentor assisted them:

Generally, I think the mentoring thing probably did not help me a great deal although I can see how it might help others that don't have much up and go or are not interested in their health. (Participant 8)

## Discussion

In Australia, despite CR programs being readily available to the general population, many do not attend (Clark *et al*. 2013). Referral rates to CR in Australia have remained low and relatively unchanged during the last two decades. Participation rates have been estimated between 10–30% (Clark *et al*. 2013) with access to secondary prevention programs less prevalent among indigenous and remotely located patients due to CR programme unavailability, distance from or travel time to secondary prevention services (De Angelis *et al*. 2008). According to the WHO, all persons with cardiac disease should have access to CR services (World Health Organization Committee 1993) and several home‐based CR programs have been shown to improve accessibility for patients unable to access CR services (Neubeck *et al*. 2009). To date, there are very few studies specifically exploring patient perceptions of home‐based CR programs, therefore, comparison with similar research is limited. This study adds to previous research reporting on the experience of patients using home‐based CR programs such as the Heart Manual (Jones *et al*. 2007; Jones *et al*. 2009) and Heart Guide Aotearoa (HGA) in New Zealand (Henwood & Moewaka Barnes 2008). Participants in our study found the AHGP enabled them to complete a programme of CR in their homes, thereby improving their access to CR even though they lived remotely from a hospital‐based programme. The AHGP also assisted them to understand, cope and adjust to life style changes after MI.

Participants in our study found it was important to rectify existing knowledge gaps about cardiac disease to understand its cause, treatment and to enhance their self‐care capabilities. A recent systematic review and meta‐analysis reported that patient education has little effect on the mortality and morbidity of patients with CHD (Brown *et al*. 2013). The same review, however, found education did improve health‐related quality of life and reduce health care use. Our participants emphasized the importance of becoming aware of their disease. This included understanding the diagnosis, treatment received and what they could do to personally lessen their risk of future events. This outcome is promising as other research has found patients lack knowledge about the causes of CHD (Stewart 2013) or understand their cardiac risk (Redfern *et al*. 2007). In common with other research where participants found home‐based CR manuals to be well organized, provide a range of topics and advice (Jones *et al*. 2007), participants in our study thought the AHG resources provided an adequate amount of information to improve their knowledge. Others report patients commonly have knowledge gaps or misconceptions (Lin *et al*. 2008) about heart disease and its impact and these must be corrected for patients to appreciate the importance of risk factor control and lifestyle modification (Goulding *et al*. 2010).

Psychological components contained in home‐based CR programs are central to their success as models of care (Lewin *et al*. 1992). In this study, participants suggested that while family support was important to them, mentors provided additional support in terms of individualized professional lifestyle advice and encouragement to promote change once discharged from hospital. Others have found that discharge education and follow up by nurses to improve patient outcomes (Koelling *et al*. 2005) and reduce anxiety related to being discharged from hospital (Carroll & Dowling 2007). According to participants, mentors methodically prepared them, their family or significant others for discharge by organizing a week to week plan of care to guide their recovery and importantly, their ongoing support help them overcome hospital discharge related anxiety.

Previous research has suggested that patients often have difficulty processing information while in hospital due to being overwhelmed by their situation (Moser *et al*. 1993). Others have found that some patients were not satisfied with primary care follow up (Radcliffe *et al*. 2009), had questions post discharge but no one to ask or received general information about after‐hospital care that was not specific to their individual circumstance (Hanssen *et al*. 2005). Researchers have previously found that patients preferred to have contact or telephone access to a health professional soon after leaving hospital (Hartford *et al*. 2002) and providing information and support via telephone after discharge significantly reduced their levels of anxiety. It appears from our findings that nurse mentors facilitating the AHGP helped participants and their families adjust and cope with their illness during their recovery period after MI.

In common with other research, some participants said their spouses exhibited signs of psychological stress, with some fearing that their partner might die. Anger, guilt, fatigue, depression and disruption of normal family dynamics are examples of other emotions reported by participant's relating to experiences occasioned by family members and widely reported in the literature (Moser & Dracup 2004). Though challenging for some nurses due to the complexities involved, growing evidence suggests that after‐hospital care of cardiac patients should also target spousal and family support (Salminen‐Tuomaala *et al*. 2013). Some suggest instead of clinicians practicing patient‐centred care (PCC), a family‐centred care (Fleury & Moore 1999) approach may reduce the stress experienced by both patients and their spouses and maximize support for both. Others suggest peer support from family members or friends remain an under used resource in patient and spousal recovery (Cotella & King 2004). Findings from a recent Finnish study suggests nurses should be aware of spousal coping strategies so they can provide information about recovery and enhance opportunities for spouses to share their experiences with their partner and significant others (Salminen‐Tuomaala *et al*. 2013).

In terms of health service delivery, the findings suggested that the AHGP used a PCC approach to facilitate patient recovery from life threatening illness. PCC refers to the individualization of patient care and participants in our study confirmed that being treated as an individual was very important to them. In PCC, healthcare professionals readily acknowledge, value and respect each patient's unique perceptions and experiences of their world (Morgan & Yoder 2012), understand them as a whole person respect their needs and preferences and importantly, involve and empower them in their own care and decision‐making (Stewart 2001).

It is clear that participants in this study felt mentors used a PCC approach to facilitate the AHGP. Interestingly, a recently published Cochrane Review of 43 randomized trials on patient‐centred approaches to healthcare delivery found that active participation did result in improvements to healthcare recipients in areas of health status, health behaviour and well‐being (Dwamena *et al*. 2012). Importantly, where PCC was supported with provider training and condition‐specific training materials, specific health behaviours showed the most improvement (Dwamena *et al*. 2012). Clearly, this aspect compares favourably with the AHGP as its resources are condition specific. While the current study asked participants about their perceptions of the AHGP, their positive experiences may actually facilitate active engagement in managing their health, which may ultimately result in better outcomes.

### Study limitations

Findings from this study may not be generalizable because each participant's individual descriptions relate to the personal meanings attached to them within the context they occurred. However, the study does provide conceptual insight into CR programs that incorporate mentorship, thus the findings may be applicable to others. The use of individual interviews with patients has several strengths including an ability to build close rapport, capacity to pace the interview to the participant's situations, allows recruitment of individuals in geographically dispersed locations and opportunities to clarify or probe for more information. However, had focus group interviews been conducted it is possible other insights may have emerged because of the group interactions. Another potential limitation of the study concerns the duration of some of the interviews. Although ranging between 10‐30 minutes, some interviews lasted only 10 minutes despite being prompted by the interviewer, presumably because the respondents had provided the information they wished to convey. This was despite purposively selecting participants. It is possible that if we had sent the interview guide to participant ahead of time, they may have had more to say. Finally, member checking of the interview transcripts were not used in this study; however, during each interview, participants’ responses were briefly summarized after each question to check that we had understood and interpreted the meaning of their experiences and recorded their verbal responses accurately. And, during each interview, participants were asked for further clarification of their responses if warranted. Also, there is debate on the merits and limitations of member checking in qualitative research (Carlson 2010).

Despite these limitations, this study does present clinicians with some insight in to what aspects of post hospital care patients recovering from cardiac related illness find meaningful. Further, the findings of this study appear to suggest that CR trained mentors may provide a viable possibility for people living in geographically remote locations to get support and have their CR needs met. Based on these findings, several areas for future research are evident. First, developing and testing various mentor training interventions may result in mentors who are able to use various techniques to better influence and support patients’ behaviour change. Second, the sustainability of both mentor based CR programs and their long‐term impact on patient behaviours and outcomes could be explored. Associated with this, cost‐effectiveness studies may help to determine if widespread use should be advocated. Finally, developing and evaluating strategies to incorporate families into the CR programme may help to improve its uptake.

## Conclusion

The findings from this study support the provision of the AHGP as a home‐based self‐help programme for patients recovering from MI who find it geographically difficult to attend hospital‐based CR programs. Patients were satisfied with the AHGP resources in terms of assisting them to understand and improve their knowledge relating to their illness and inform them of unhelpful lifestyle behaviours. The mentor–patient relationship, based on principles of individualized care was highly valued by participants. Mentors were perceived to be integral to the success of the programme through the provision of timely information, ongoing psychosocial support and lifestyle advice to patients during their recovery from MI.

## Conflict of interest

All authors have agreed to the final version of this paper and have all made contributions to its writing. No conflict of interest has been declared by the authors.

## Author contributions

All authors have agreed on the final version and meet at least one of the following criteria [recommended by the ICMJE (http://www.icmje.org/recommendations/)]:

- substantial contributions to conception and design, acquisition of data or analysis and interpretation of data;
- drafting the article or revising it critically for important intellectual content.
